# Effect of continuous furosemide infusion on outcome of acute kidney injury

**DOI:** 10.12669/pjms.35.3.1012

**Published:** 2019

**Authors:** Jie Ni, Hui Jiang, Fang Wang, Long Zhang, Dujuan Sha, Jun Wang

**Affiliations:** 1 *Jie Ni, Department of Emergency, Nanjing Drum Tower Hospital, The Affiliated Hospital of Nanjing University Medical School, No. 321 Zhongshan Road, Gulou District, Nanjing 210000, Nanjing Province, P. R. China*; 2 *Hui Jiang, Department of Neurology, Jiangsu Province Hospital of Traditional Chinese Medicine, Affiliated Hospital of Nanjing University of Traditional Chinese Medicine, Nanjing 210000, China*; 3 *Fang Wang, Department of Emergency, Nanjing Drum Tower Hospital, The Affiliated Hospital of Nanjing University Medical School, No. 321 Zhongshan Road, Gulou District, Nanjing 210000, Nanjing Province, P. R. China*; 4 *Long Zhang, Department of Emergency, Nanjing Drum Tower Hospital, The Affiliated Hospital of Nanjing University Medical School, No. 321 Zhongshan Road, Gulou District, Nanjing 210000, Nanjing Province, P. R. China*; 5 *Dujuan Sha, Department of Emergency, Nanjing Drum Tower Hospital, The Affiliated Hospital of Nanjing University Medical School, No. 321 Zhongshan Road, Gulou District, Nanjing 210000, Nanjing Province, P. R. China*; 6 *Jun Wang, Department of Emergency, Nanjing Drum Tower Hospital, The Affiliated Hospital of Nanjing University Medical School, No. 321 Zhongshan Road, Gulou District, Nanjing 210000, Nanjing Province, P. R. China*

**Keywords:** Acute kidney injury, Furosemide, Pulmonary edema

## Abstract

**Objective:**

To evaluate the clinical effects of continuous intravenous infusion with high-dose furosemide on early acute kidney injury (AKI) complicated with acute lung edema.

**Methods:**

Ninety patients who had been treated by furosemide at routine dose for 12 hour but with unsatisfactory outcomes were selected and subjected to continuous intravenous infusion with high-dose furosemide. The dose was adjusted according to hourly urine output. Serum levels of urea nitrogen, creatinine and potassium, pH, oxygenation index and mechanical ventilation time before and 6, 12, 24, 48 and 72 hour after treatment were compared.

**Results:**

The urine outputs before and 6, 12, 24, 48 and 72 hour after treatment were (10.71 ± 1.81), (164.52 ± 21.42), (189.71 ± 29.61), (181.33 ± 23.52), (176.82 ± 24.80) and (164.52 ± 18.91) ml/h respectively. Compared with data before treatment, the serum levels of urea nitrogen, creatinine and potassium significantly decreased while pH and oxygenation index significantly increased after six hour of treatment (P<0.05). After treatment, the kidney functions of 80 patients (88.9%) were completely recovered, without obvious adverse reactions.

**Conclusion:**

For patients with early AKI complicated with acute pulmonary edema who cannot be cured by diuretic agent at routine dose, high-dose furosemide increases urine output and improves success rate.

## INTRODUCTION

Acute kidney injury (AKI) is a common clinical syndrome endangering human health and leading to inpatient death. Until 2013, about 13 million people suffered from AKI each year globally, of whom 1.7 million people died.^1^ AKI is pathologically typified by proximal tubular injury, edema, necrosis and formation as well as epithelial cell apoptosis, accompanied by severe disorders of the internal environment due to rapidly damaged kidney function. Its clinical manifestations mainly include oliguria or anuria, azotemia, hyperkalemia and metabolic acidosis, seriously endangering human life in case of ineffective rescue.^2^ Owing to the lack of effective therapies, over 50% of AKI patients cannot recover,^3^ and progress to chronic kidney disease instead. Finally, end-stage renal disease occurs, which heavily burdens their families and the society.^4^

As a potent diuretic agent, furosemide can enhance glomerular filtration, and inhibit the reabsorption by distal and proximal convoluted tubules to alleviate water poisoning. Although AKI patients complicated with oliguria have been successfully treated by high-dose furosemide, the therapeutic effects remain controversial.^5^ In this study, we assessed the effect of high-dose furosemide on patients with early AKI complicated with acute pulmonary edema, aiming to provide valuable clinical evidence for mitigating the symptoms and improving the prognosis.

## METHODS

### Baseline clinical data

This study was a retrospective clinical trial. It was approved by the ethics committee of our hospital, and written consent has been obtained from all patients. Ninety patients with early AKI complicated with acute pulmonary edema, who had been treated by furosemide at routine dose for 12 hour but with unsatisfactory outcomes in our hospital from June 2012 to June 2016, were selected. They consisted of 48 males and 42 females aged 45~81 years old, (61.11 ± 12.28) on average. The symptoms of 70, 14 and 6 cases were caused by coronary artery disease-induced heart failure, cardiopulmonary resuscitation and sepsis respectively.

### Inclusion criteria

1. In accordance with the diagnostic criteria of RIFLE grading for early AKI:^6^ Obvious renal function decline within seven day, urine output of <0.5 mL/kg·h^-1^ for 6-24 hour or serum creatinine elevation of 1.5- to 3.0-fold (or 26.4~354.0 μmol/L), and GFR of <60 mL/min.
2. Complication with severe respiratory failure or acute pulmonary edema which required mechanical ventilation and emergency reduction of volume load.
3. Treatment with furosemide at routine dose (100 mg/d) for 12 hour, but with unsatisfactory outcomes.
4. Refusal of continuous renal replacement therapy (CRRT) by family members.
5. With stable hemodynamics, mean arterial pressure (MAP) of >65 mmHg and central venous pressure of 12~18 mmHg.
6. With written consent from all patients and family members.

### Exclusion criteria

Patients who had chronic renal failure, allergy to furosemide, obstructive kidney injury, hypovolemia or septic shock, or required CRRT. RIFLE grading criteria for AKI: Risk: Kidney function declined within seven days, and serum creatinine level increased 1.5-fold or by ≥26.4 μmol/L or urine output remained ≤0.5 ml·kg^-1^·h^-1^ for 6 h; injury: serum creatinine level doubled or urine output remained ≤0.5 ml·kg^-1^·h^-1^ for 12 h; failure: serum creatinine level increased 3-fold or by ≥354 μmol/L, or urine output remained ≤0.5 ml·kg^-1^·h^-1^ for 24 h or anuria remained for ≥12 hour.

### Methods

Based on routine multiple organ support therapy, all patients were administered with high-dose furosemide. In detail^7^, they received continuous intravenous infusion with 1~2 mg/min furosemide, and the dose was adjusted according to hourly urine output (target: 2~4 ml·kg^-1^·h^-1^), systemic volume, tissue anoxia, MAP and oxygenation index.^8^ The patients were observed for 72h. During treatment, the changes of electrolyte levels and vital signs were monitored, and electrolyte disorders were timely corrected. Furosemide was no longer used under the following circumstances: Urine output did not change after 6 h of infusion with 1~2 mg/min furosemide; renal replacement therapy was used; admission in the ICU was not required; patients died; kidney function recovered; severe treatment-related adverse reactions occurred.

### Observation indices

Serum levels of urea nitrogen, creatinine and potassium, pH, oxygenation index and mechanical ventilation time before and 6, 12, 24, 48 and 72 h after treatment were compared. Adverse reactions such as deafness and tinnitus were recorded.

### Recovery criteria for kidney function

Compared with the data before treatment, serum creatinine level dropped by 10% and urine output remained ≥1.0 ml·kg^-1^·h^-1^ for at least 24 h. The RIFLE grade decreased by one.^9^

### Statistical analysis

All data were analyzed by SPSS 17.0. The categorical data were expressed as mean ± standard deviation (x ± s). The data before and after treatment were compared by one-way analysis of variance. Pairwise comparisons were conducted by the LSD method. P<0.05 was considered statistically significant.

## RESULTS

The urine outputs before and 6, 12, 24, 48 and 72 h after treatment were (10.71 ± 1.81), (164.52 ± 21.42), (189.71 ± 29.61), (181.33 ± 23.52), (176.82 ± 24.80) and (164.52 ± 18.91) ml/h respectively (Table-I).

**Table-I:** Urine outputs after using furosemide for different times.

Time	Mean furosemide dose (mg/h)	Mean urine output (ml/h)
Before treatment	-	10.71±1.81
6 h after treatment	106.41±20.92	164.52±21.42
12 h after treatment	71.4±10.31	189.71±29.61
24 h after treatment	22.81±9.82	181.33±23.52
48 h after treatment	3.21±0.82	176.82±24.80
72 h after treatment	-	164.52±18.91

The serum levels of urea nitrogen, creatinine and potassium all dropped after six hours of treatment compared with those before treatment, which significantly decreased with extended time (P<0.05). The pH and oxygenation index after 6 h of treatment both significantly rose compared with those before treatment (P<0.05), which also significantly increased with prolonged time (P<0.05) (Table-II).

**Table-II:** Observation indices before and different times after treatment.

Time	Before treatment	6 h after treatment	12 h after treatment	24 h after treatment	48 h after treatment	72 h after treatment	P value
Serum urea nitrogen (mmol/L)	27.81±1.82	23.78±1.89*	19.18±1.98*	12.19±1.54*Δ▲	10.45±1.34*Δ▲	9.15±1.45*Δ▲☆★	0.000
Serum creatinine (μmol/L)	472.45±43.09	413.45±43.18*	397.18±19.15*Δ	362.19±24.59*Δ▲	323.87±15.49*Δ▲☆	258.19±34.15*Δ▲☆★	0.000
pH	7.12±0.06	7.17±0.02*	7.22±0.03*Δ	7.25±0.04*Δ▲	7.32±0.04*Δ▲	7.40±0.03*Δ▲☆★	0.000
Oxygenation index (mmHg)	100.43±21.32	124.51±12.43*	153.23±17.19*Δ	195.14±26.58*Δ▲	251.42±26.19*Δ▲☆	313.78±24.19*Δ▲☆★	0.000
Serum potassium (mmol/L)	5.61±0.27	5.32±0.12*	5.00±0.17*Δ	4.68±0.19*Δ▲	4.31±0.18*Δ▲☆	4.22±0.22*Δ▲☆	0.000

Comparison with before treatment,

* P<0.05: comparison with 6 h after treatment,

Δ P<0.05: comparison with 12 h after treatment,

▲ P<0.05: comparison with 24 h after treatment,

☆ P<0.05: comparison with 48 h after treatment,

★ P<0.05.

After treatment, the kidney functions of 80 patients (88.9%) were completely recovered, and dyspnea was evidently alleviated. Chest X-ray disclosed that pulmonary edema was markedly remitted, no longer requiring mechanical ventilation. The average mechanical ventilation time was (36. 34 ± 10. 81) hour.

During treatment, no patient suffered from adverse reactions such as tinnitus and deafness. Meanwhile, the hemodynamics remained stable.

## DISCUSSION

Glomerular filtration rate in acute renal injury suddenly decreases, which clinically shows the continuous increase of serum creatinine, decreased urine output, along with electrolyte imbalance and the production of nitrogenous products. The incidence of AKI in critically ill patient’s ranges from 36% to 67%.^10^ Despite advances in organ support technology, the prognosis of AKI patients in ICU is still not optimistic. RRT is required for 5% to 6% of patients with AKI, which, however, is expensive and cannot be used universally. Therefore, it is of great significance to explore the drug therapy of AKI.

Oliguric AKI usually has more severe renal damage, with a worse prognosis than non-oliguric kidney injury, which is often complicated by severe metabolic acidosis, hyperkalemia and water intoxication, and even endangers the lives of patients. Studies have shown that the positive balance of fluids is closely related to the mortality of AKI patients.^11^ Therefore, trying to control the fluid load and striving to restore urine output and expecting the change from oliguric renal failure to non-oliguric renal failure is of great importance in controlling the occurrence of severe complications and improving the prognosis of patients. There is currently considerable controversy over the use of furosemide. Most studies suggest that diuretics cannot improve the prognosis of AKI patients, and may even worsen kidney damage, so diuretics are not recommended.^12^,^13^

However, for AKI patients with excessive volume overload, furosemide can be used to alleviate excessive volume overload, improve pulmonary edema caused by AKI, reduce the frequency of RRT application and treatment costs, shorten hospitalization time, alleviate injury to kidney function, and help patients go through dangers.^14^,^15^ Thus, furosemide is widely used in patients with early AKI and acute pulmonary edema who cannot be performed with RRT for various reasons. However, AKI patients who are refractory to routine doses of furosemide often experience clinically severe complications, such as pulmonary edema, metabolic acidosis, and hyperkalemia. If the patients are not treated in a timely and effective manner, their life will be threatened. Accordingly, it is imminent for patients to escape life-threatening risks as soon as possible, so CRRT is the best treatment for these patients, but many families cannot afford its high costs. The application of high-dose furosemide may provide a therapeutic regimen for the treatment of life-threatening AKI patients.

There are relatively few reports on the use of high-dose furosemide. Felker et al. reported that the use of furosemide in AKI patients caused by heart failure can reach 4000 mg/d;^16^ there have also been reports that continuous injection of high-dose furosemide is both effective and safe for the volumetric therapy of hemodynamically unstable children after cardiac surgery.^17^ In this study, patients with early AKI complicated with acute pulmonary edema who were refractory to conventional doses of furosemide were given a large dose of furosemide (1 to 2 mg/min) for continuous venous injection. The results showed that the patient’s urine volume obviously increased six hours after treatment, reaching 2 to 4 ml • kg^-1^ • h^-1^ target urine volume; the levels of blood urea nitrogen, serum creatinine and serum potassium decreased compared with before treatment; pH and oxygenation index increased compared with before treatment; the average mechanical ventilation time was (36.34 ± 10.81) h; 88.9% of the patients had renal function recovery and were successfully removed from equipment, with their prognosis improved.

## CONCLUSION

In summary, continuous intravenous infusion of high-dose furosemide provides an effective treatment for patients with early AKI and acute pulmonary edema who are unwilling to receive RRT. Meanwhile, large doses of furosemide can correct electrolyte and acid-base balance disorders in some patients, improve the internal environment of the body, and reduce blood urea nitrogen and serum creatinine levels in a small proportion of patients, at least not aggravate renal impairment. However, due to the small number of cases in this study, there was no follow-up after discharge. Hence, whether high-dose furosemide treatment affects the long-term prognosis of AKI patients still needs further study and discussion.

### Authors’ Contributions

**JN, HJ, FW & LZ** performed this study, analyzed clinical data and prepared this manuscript.

**DS & JW** designed this study and revised this manuscript.
